# Underrated past herbivore densities could lead to misoriented sustainability policies

**DOI:** 10.1038/s44185-022-00005-z

**Published:** 2023-01-06

**Authors:** Pablo Manzano, Guillermo Pardo, Moustapha A. Itani, Agustín del Prado

**Affiliations:** 1 Global Change and Conservation Lab, Organismal and Evolutionary Biology Research Programme, Faculty of Biological and Environmental Sciences, University of Helsinki, Helsinki, Finland; 2 Helsinki Institute of Sustainability Science (HELSUS), Faculty of Biological and Environmental Sciences, University of Helsinki, Helsinki, Finland; 3 Basque Centre for Climate Change (BC3), Leioa, Spain; 4 Ikerbasque—Basque Foundation of Science, Bilbao, Spain

**Keywords:** Ecology, Environmental sciences, Agriculture, Forestry

## Abstract

Knowing the carrying capacity of the Earth’s grazed ecosystems, and the relevance of herbivory, is important for many scientific disciplines, as well as for policy. Current herbivore levels are estimated to be four to five times larger than at the Pleistocene–Holocene transition or the start of the industrial revolution. While this estimate can lead the general public and the scientific community to predict severe, widespread environmental impacts by livestock in terms of deforestation, biodiversity loss, and climate change, it ignores the inherent uncertainty of such calculations. We revise the evidence published during the last decade regarding Late Pleistocene herbivore abundance, along with contemporary and some pre-industrial data on herbivore density in grazed ecosystems. Both Late Pleistocene and pre-industrial herbivore levels are likely to be consistently higher than what has generally been assumed, confirming increasing awareness on the importance of herbivory as a widespread ecological process. We therefore call for more refined research in this field to have the reliable baselines currently demanded by society and policy. These baselines should orient sound action toward policies on biodiversity conservation, ecosystem restoration, food systems, and climate change.

## Introduction

Estimating the total carrying capacity of the Earth’s terrestrial ecosystems to host mammalian herbivores is a key question for multiple fields of sustainability science, a transdisciplinary field with elements from sociology, economic and environmental sciences where herbivore density greatly conditions aspects related to ecology, climate science, and agronomy. It carries important implications for understanding the relative importance of herbivory, fire, and litter-decomposing microbes in recycling matter and shaping ecosystems, and thus for establishing the real extent of naturally occurring forests versus alternative states that display more open ecosystems^1^. Knowing the potential herbivore pressure is also important to establish livestock loads that preserve habitat integrity and avoid land degradation or biodiversity loss. But it also allows to establish greenhouse gas emission baselines that correspond to natural ecosystem fluxes, thereby allowing to improve the estimates of anthropogenic greenhouse gas emissions^2^. For global warming analyses, it is important to refine which emissions from grazing livestock systems could be alternatively assigned to natural sources, as food system transitions toward more intensive livestock systems are seen as a powerful mitigation strategy^3,4^.

## Estimates of natural herbivore abundance

Recently, Bar-On et al.^5^ published an estimate of the total biomass on Earth, widely cited by other academic publications. Most notably, it included an estimate of the current mammal herbivore biomass in the planet, including humans, domestic livestock, and wild mammals as separate categories. They compared them with Barnosky’s 2008 estimate^6^ of wild mammal biomass before the Quaternary mass extinction, to conclude that humans have managed to increase the biomass of large land mammal species—including themselves—by a factor of 4.5 (from ≈0.04 Gt C to ≈0.17 Gt C). While Bar-On et al.^5^ warn that pre-human values “are very difficult to estimate accurately”, and Barnosky^6^ himself admitted using what he regarded as “the most reasonable input parameters”, the interpretation that many media and environmental advocacy organizations have done about these values shows that their inherent uncertainty has been largely ignored. We here want to review the available evidence published on the subject since 2008, coming from different academic fields, that may help not only understanding realistic potential herbivore loads of ecosystems but also orienting future research.

## Late Pleistocene evidence

In 2016, Smith et al.^7^ related methane concentration in air bubbles trapped in ice cores to the Quaternary megafaunal biomass at different times of the Holocene. The values they estimated for the Late Pleistocene are very different, however. While Barnosky^6^ interpreted it to be ca. 2 × 10^11 ^ kg, the data used by Smith et al^7^ add to a value of ca. 8 × 10^11 ^ kg (Table 1). The analysis of Barnosky^6^ is largely exploratory, expressing that the result “depends on assumptions that were explored in the sensitivity tests”, which derive from what he regards “as the most reasonable input parameters”, but he warns that “additional refinements would be desirable […] beyond the scope of this initial work”. The numbers given by Smith et al.^7^, however, are consistent with factual, historic CH_4_ concentrations compared in the same paper, and should therefore be interpreted as more robust.

**Table 1:** Density of herbivores from locations situated in different conventionally assumed biomes^18^ but belonging to either Open Ecosystems^11^ or the Mammoth Steppe^8^.

#	Location	Biome	Wild herbivore density	Current livestock density	Of which ruminants	Baseline period	Source (wild herbivore baseline)	Source (current livestock)
1	Alaska & Northeastern Siberia	Mammoth steppe	8.8–10.5	0.1	0.1	Pleistocene	^12^, data from refs. ^8^ and ^52^	Mean value of both regions
2	Great Britain	Temperate wood pasture	≥12.5	33.3	28.3	Last InterGlacial	^12^, data from ref. ^53^	Mean value from overlap with Open Ecosystems^11^
3	Spain	Temperate wood pasture	10.7	15.2	8.8	Pre-industrial	^25^	
4	Europe, North America	Temperate wood pasture	11–18.7	12.4	8.9	Present-subjected to rewilding	^12^, data from diverse papers	
5	North American prairie	Temperate grassland	10.3	13.1	11.2	Pre-industrial	^27^ with distribution area data from ref. ^26^	
6	Uruguay; Buenos Aires, Argentina	South American Pampas	10.35	27.5	27.0	Late Pleistocene & Early Holocene	^54^	
	Africa	Tropical savanna	6.8–7.8	n.a.	n.a.	Present-protected areas	^19^ excl. low NPP areas	n.a.
	Africa	Grasslands & deserts	3.2	n.a.	n.a.	Present-protected areas	^19^—low NPP	n.a.
7	Akagera	Tropical savanna	10.9	11.3	10.8	Present-protected areas	^19^	Estimated as the mean value of the surrounding area (10,000 km^2^)
8	Amboseli	Tropical savanna	14.2	9.7	9.5			
9	Bunyoro North	Tropical savanna	13.3	8.0	7.6			
10	Kaputei Plains	Tropical savanna	21.3	11.6	11.2			
11	Manyara	Tropical savanna	15.3	10.8	10.7			
12	Masai Mara	Tropical savanna	12.1	23.1	23.0			
13	Ngorongoro Crater	Tropical savanna	14	9.6	9.5			
14	Queen Elizabeth	Tropical savanna	11.8	12.2	10.9			
15	Serengeti	Tropical savanna	11.6	13.5	13.4			
16	Simanjiro	Tropical savanna	8.5	8.2	8.1			
17	Selous	Tropical savanna	9	1.3	1.2			
18	Katavi	Tropical savanna	7.8	2.4	2.4			
19	Hluhluwe iMfolozi	Tropical savanna	9.2	8.8	8.7			
20	Sabie River Kruger NP	Tropical savanna	9.1	5.1	5.1			

Animal densities are expressed in tonnes per km^2^. Current livestock densities are sourced from ref. ^51^.

Conservative estimates for the ancient Siberian steppe ecosystem, also called Mammoth Steppe^8^, report a herbivore density at the beginning of the Holocene of ca. 10 tonnes/km^2^. Such herbivore density would have been higher in more southern latitudes of the same biome. In any case, if taken such value as the mean density of the whole biome (with a maximum Pleistocene extent of ca. 30 million km^2^)^9,10^, the resulting value is 3 × 10^11 ^ kg, exclusively for such periarctic biome. This exceeds Barnosky’s^6^ estimate for all terrestrial ecosystems combined (148 million km^2^) by 50%, even if the Mammoth Steppe is not the only biome supporting large herbivore densities. Some of remaining terrestrial areas at that time such as Antarctica, the Tibetan plateau, or the areas covered by the Laurentide’s ice sheet would have had a nil or negligible natural herbivore load. But widespread Holocene biomes usually considered closed forests with low herbivore density have shown to be able to support rich, abundant herbivore guilds under alternate ecosystem states^11^.

## Calculations from contemporary grazing ecosystems

Further estimates (^12^; Table 1) show that herbivore densities per km^2^ currently observed in other grazing ecosystems around the world are within the same range as the Mammoth Steppe densities^8^. It should be noted that grazing ecosystems displaying migratory patterns, such as, e.g., the Serengeti, display dominating bottom-up regulations because of the predator escape opportunity brought by migration. Also, a more diverse herbivore guild will be able to exploit ecological niches more efficiently, giving way to a higher herbivore biomass density. As an example, the equilibrium red deer density in protected grasslands in semiarid Spain is 33 deer/km^2^ (see ref. ^13^), equivalent to a biomass density of ca. 4 tonnes/km^2^. These are non-migratory systems and hence with a lower carrying capacity of the ecosystem, with a simplified herbivore guild consisting just of browsers – grazers such as aurochs or wild horses becoming extinct long ago. Higher wild or restored^14^ herbivore loads are therefore to be expected.

**Fig. 1:**
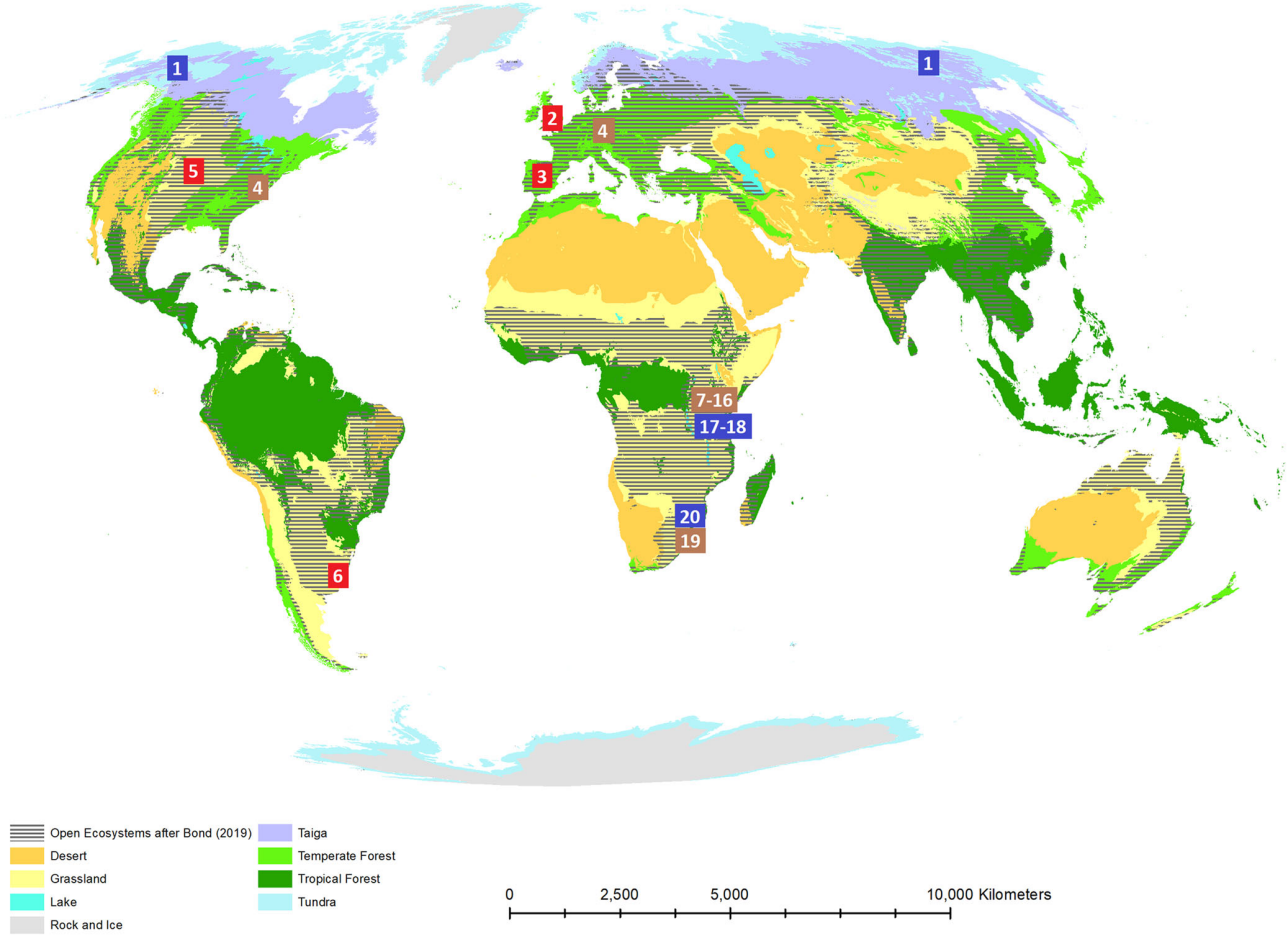
Overlap between conventionally assumed biomes^18^ and Open Ecosystems^11^. High herbivore densities, associated with either Open Ecosystems, or the Mammoth Steppe^8^, irrespectively of their biome type, are numbered according to Table 1. Locations, where livestock density is notably greater than the baseline herbivore density, are labeled in red; if notably lower, in dark blue; and if no deviation is observed, in brown. World biome map layer sourced from Esri GeoInquiries (World Wildlife Fund, Esri Data and Maps), licensed under Creative Commons (BY-NC-SA). Open Ecosystems layer source: own work.

An estimate can be done of the total extent of such grazing ecosystems. For this purpose, we first added the current extent of the taiga biome which would largely fall into the rather broad climatic envelope of the Mammoth steppe (^8^; see ref. ^9^ for extent), to the areas with climate potential for closed forests but held open by herbivores and fire^11^. Such open ecosystems often overlap with ecosystems considered to be closed-canopy forests (Fig. 1), similar to the case of taiga vs. Mammoth steppe. To calculate the resulting added area we replicated the calculations in by Bond^15^ by using WorldClim 2 climatic data^16^. The area calculation was performed by utilizing the World Cylindrical Equal Area projection using ArcGIS software^17^. We obtained a total area of 48.57 million km^2^, to which we added 13.5 million km^2^ from the taiga biome^18^, resulting in a total of 62.1 million km^2^.

Open Ecosystems sensu Bond^11,15^ consistently host herbivore densities of at least 10 tonnes of herbivores/km^2^ according to literature sources published after Barnosky^6^, as summarized in Table 1. For Africa, Fløjgaard et al.^19^ estimate a density of 6.8 and 7.8 tonnes/km^2^ at high and mid net primary productivity levels, corresponding to African Open Ecosystem areas. However, such average values are burdened by the heavy fragmentation of agricultural areas that surround National Parks in West Africa, and that also hinder mobile pastoralism^20^, by the encroachment of grazing resources by intruding pastoralists^21^, or just by the impossibility to perform migrations. Specific protected areas mentioned by Fløjgaard et al.^19^ having large areas or surrounding landscapes that are permeable enough to allow large-scale herbivore migrations are also listed in Table 1 and Fig. 1. It should be noted that the areas with higher densities are concentrated in East Africa (numbers 7–16 in Table 1 and Fig. 1), probably related to the prevalence of pastoralist economies in the area thanks to the advantage for milk economies^22^ and the coexistence that has observed to be possible between wild herbivores and pastoralism in the area^23^. A large landscape matrix preserved through pastoralist uses would explain such higher herbivore densities in the region. Thus, we used the value of 10 tonnes/km^2^ in our analysis, which yield a global potential herbivore biomass of 6.2 × 10^11^ kg in Open Ecosystems.

Drier treeless areas, known to host large wild herbivore herds (e.g., Saiga antelope in Mongolia and Kazakhstan; wild yak in the Tibetan Plateau) or rainforest areas known to host lower densities of megaherbivores (e.g., forest elephants in the Congo forests, or Asian elephants and diverse rhino species in Southeast Asia) have a lower herbivore carrying capacity, yet they occupy a very large area—the remaining 86.8 million km^2^ of emerged land. Subtracting the barren lands devoid of herbivores of Greenland and Antarctica (a total 15.9 million km^2^), we applied a conservative estimate of 1 tonne of herbivores per km^2^ for the remaining lands (70.9 million km^2^) would yield an additional 7 × 10^10 ^ kg. We consider this value to be conservative in light of the values reported by e.g., Fløjgaard et al.^19^, where African low productivity protected areas are reported to have an average value of 3 tonnes of herbivores per km^2^.

Together, Open Ecosystems and lower productivity areas yield a global biomass of ca. 7 × 10^11 ^ kg. This is a much higher value than Barnosky’s^6^ estimate. It would also be consistent with the 8 × 10^11 ^kg Smith et al.’s estimate^7^, confirming their interpretation that current, livestock-dominated, herbivore-mediated methane emissions are not substantially exceeding the natural baseline emissions attributable to natural flows^2^. It also confirms Barnosky’s^6^ interpretation of primary productivity captured by natural herbivory overwhelmingly being shifted to livestock, but with an important nuance. Industrial livestock production would have exceeded natural herbivore herd size only by a fraction (Table 2).

**Table 2:** Comparison of approximate global herbivore biomass at the Pleistocene/Holocene transition and in contemporary time according to different studies discussed in this paper.

Source	Late Pleistocene	Modern pre-industrial	Today
Barnosky^6^	2 × 10^11 ^kg	2 × 10^11 ^kg	9.5 × 10^11 ^kg
Smith et al.^7^	8 × 10^11 ^kg	2.8 × 10^11 ^kg	9.5 × 10^11 ^kg
Bar-On et al.^5^	n.a.^†^	n.a.	7.4 × 10^11 ^kg

*n.a.* does not apply.

^†^Value taken from Barnosky^6^.

## Pre-industrial herbivore densities

A further caveat appears for the pre-industrial baseline of herbivore densities assumed by both Barnosky^6^ and Smith et al.^7^. A decreasing phase since the start of the Holocene (corresponding to wild megafaunal declines) is followed by an increasing phase (corresponding to increases in grass-fed livestock), until reaching a point of equilibrium at the time of the Industrial Revolution. In both papers, it is argued that the values are the same at both points (start of the Holocene and start of the Industrial Revolution) because of similar, intense use of available grazing resources. At a time when societies holding large amounts of livestock were already widespread in Europe, the Indian subcontinent, China, the Asian steppes, the Sahel or the Eastern and Southern African savannas, and large herds having already developed in extensive parts of Latin America, their estimates for pre-industrial livestock levels seem low (Table 2). A drastic reduction of primary production in grazed ecosystems since the end of Pleistocene would be difficult to explain, especially considering livestock densities in areas with available data. Spain has an exceptionally detailed accounting of livestock abundance in pre-industrial times, not found, to our knowledge, in any other country, motivated by the statistical investigation of the Marquess of Ensenada^24^. The combined data of the grazed area and livestock abundance^25^ yield a density of 10.7 tonnes/km^2^ for 1750, comparable to the ones mentioned for a diverse array of grazed ecosystems (Table 1). Further available pre-1800 information, albeit less precise, exists on the plain bison, whose range is estimated at 3.1 million km^2^ (see ref. ^26^) and for which the medium population size and average body weight used by^27^ yield a density of 10.3 tonnes/km^2^—a value, again, consistent with other grazing ecosystems discussed here (Table 1). Further research is needed to estimate the numbers of pre-industrial livestock at a global scale, but it becomes clear that previous interpretations should be handled with extreme care.

## Policy recommendations

The information reviewed in this paper confirms recent results^19^ that point to herbivore density being way below natural levels in protected areas outside Africa. Research in the last decade shows a more important role of mammal herbivory in shaping the Earth’s ecosystems, both in terms of extent of grazed ecosystems, and of higher natural herbivore densities. A more accurate consideration of both factors can improve the quality of ecological restoration initiatives both in terms of assuming the real role of herbivores in natural habitats^14^ and in not overspreading closed-canopy forests^28,29^. Nuancing such insights has important implications, e.g., for assumptions of wilderness areas^30,31^ and whether defaunation by humans may have affected their primeval state, or for calculating the potential capacity of carbon fixation by forests, which may be overstated, as e.g., in ref. ^32^, and may lead to reconsidering calls for afforesting grazed areas^4^.

It is important to nuance the ecological function of domestic herbivores when reintroduction of wild herbivores for ecological restoration is economically or socially unfeasible. The “intermittent nature of herbivory in natural systems”, mentioned^33^ as a key factor for maintaining sustainability of grazing, is also present in mobile pastoralist systems. They provide important functions in terms of tree regeneration^34^, seed dispersal or pollinator facilitation^35^ while achieving higher productivity than sedentarized systems^36,37^, yet they constitute a dwindling system due to inadequate policy and legistation^38^. Conversely, the current trend towards intensification of livestock production in high-income countries^39^ that rural development policies in low-income countries also aim for^40^, results in undergrazed landscapes that are prone to biodiversity loss and to wildfires^41^, as well as in severe impacts related to high livestock densities in intensified farms^42^. In a telecoupled global livestock production system^43^, the current abundance or distribution pattern of livestock tells hence little about the ecological role that the global livestock herd is currently having.

Current livestock densities show important mismatches with baseline natural herbivore densities in some regions (Table 1). For example, Great Britain or Spain hosting particularly large national herds due to external inputs from other continents^44^, while the boreal taiga areas in Alaska and Northeast Siberia currently show very low herbivory levels that are allegedly behind the loss of the Mammoth Steppe^8^. “Excess” livestock originates not only in monogastrics that are more favored by transcontinental fodder imports^44^, as seems to be happening in Spain or the North American prairie, but also in ruminants that may be receiving inputs that originate beyond their grazing systems, such as in Great Britain or the humid Pampas (Table 1). The high potential for sustainable livestock production pointed out by the high natural densities of wild herbivores (Table 1) shows, however, that a move towards sustainable livestock production that mimics the ecological functionality of wild herbivory should be able to provide significant amounts of animal-sourced products at an acceptable environmental impact.

Livestock, as an important part of the current global food system, needs changes to increase its sustainability^45^. This could be partly achieved both by re-designing local livestock systems, through the introduction of regenerative practices that mimic wild herbivore grazing and their movements, without changing livestock abundance^46^, and through increasing livestock densities in underoccupied areas, such as the taiga biome^8^, while decreasing them in overoccupied^44^ ones (Fig. 1). Such redistribution of livestock is also advisable at smaller scales, as revealed by the increasing wildfire problem in undergrazed areas, as happens in Spain^41^. Reduced livestock population in some African areas revealed by Table 1 should not imply a necessity to increase livestock there, for they may displace local wild herbivore populations that live outside protected areas, whose value is high for conservation and for ecosystem functionality^47^ and whose numbers are already very reduced at the global scale^5^.

A better understanding of past and current methane emissions from wild and domestic herbivores also has implications for climate policy, particularly regarding free-ranging livestock systems^2^. Beyond methane, effects of wild herbivores on climate through soil carbon^33^, such as the oxidation of soil carbon exposed by digging herbivores^48^, or higher widespread risk of fire^49^, should be also accounted for. Reliable baselines would help discern how much of the legacy of methane emissions from global livestock has been led by a shift between natural to anthropogenic (livestock) emissions. Such baselines are likely to greatly nuance current calls for a shift into plant-based diets, as in ref. ^50^. Further research should better integrate knowledge from vegetation and animal science to provide more detailed estimates of natural herbivore loads in a range as wide as possible of terrestrial ecosystems, especially accounting for differences in soil fertility. Better data would help establishing robust baselines to better understand past ecosystems and to set solid foundations for contemporary climate and biodiversity policies.

### Reporting summary

Further information on research design is available in the Nature Research Reporting Summary linked to this article.

### Supplementary information

Reporting Summary

## Data Availability

All data generated or analyzed during this study are included in this published article.
